# The role of kidney biopsy in immune checkpoint inhibitor nephrotoxicity

**DOI:** 10.3389/fmed.2022.964335

**Published:** 2022-08-10

**Authors:** Emily M. Moss, Mark A. Perazella

**Affiliations:** ^1^Department of Internal Medicine, Yale University School of Medicine, New Haven, CT, United States; ^2^Division of Nephrology, Department of Internal Medicine, Yale University School of Medicine, New Haven, CT, United States; ^3^Veterans Affairs Connecticut Healthcare System, Veterans Health Administration, West Haven, CT, United States

**Keywords:** acute kidney injury, kidney biopsy, immune checkpoint inhibitor, drug induced kidney injury, interstitial nephritis

## Abstract

Immune checkpoint inhibitors, medications that boost host immune response to tumor cells, are now at the forefront of anti-cancer therapy. While efficacious in the treatment of patients with advanced cancer, immune checkpoint inhibitors can lead to serious autoimmune side effects involving any organ in the body. Immune checkpoint inhibitor nephrotoxicity is an increasingly recognized cause of acute kidney injury in patients with cancer. This review discusses the clinical and histopathologic diagnosis of immune checkpoint inhibitor nephrotoxicity, highlighting the need for more reliable non-invasive diagnostic testing. We focus on the controversy surrounding the role of kidney biopsy in diagnosis and management of suspected immune checkpoint inhibitor toxicity with inclination toward pursuing kidney biopsy in certain outlined circumstances. Finally, we briefly discuss treatment of immune checkpoint inhibitor nephrotoxicity and the decision to re-challenge immunotherapy in patients who experience these adverse events.

## Introduction

Immune checkpoint inhibitors (ICPIs) have revolutionized anti-cancer treatment since their introduction in 2011 (1, 2). Principles of immune checkpoint blockade in cancer therapy grew from early mouse models showing antibodies against T-cell and tumor co-inhibitor signals cytotoxic T-lymphocyte-associated protein 4 (CTLA-4), programmed cell death protein 1 (PD-1) or programmed cell death ligand 1 (PD-L1) could induce tumor regression (3–6). Humanized monoclonal antibodies to these targets (ipilimumab, anti-CTLA-4; pembrolizumab and nivolumab, anti-PD-1; atezolizumab, avelumab and durvalumab, anti-PD-L1) were then studied in patients with advanced melanoma and non-small cell lung cancer, demonstrating a significant survival benefit (1, 7–10). After initial approval for metastatic melanoma, ICPIs are now approved by the Food and Drug Administration (FDA) for treatment of over 21 cancers (11).

By releasing T-cells from signaled arrest, ICPIs allow for upregulation of immune surveillance and promotion of anti-tumor activity. Widespread disinhibition of T-cells also leads to a unique set of autoimmune side effects, termed immune-related adverse events (irAEs). Immune-related adverse events can affect any organ in the body, including most commonly the skin, gastrointestinal tract, pulmonary and endocrine systems (12, 13). Renal consequences such as acute kidney injury (AKI), various glomerulopathies and electrolyte abnormalities occur but are less frequent. In this review, we explore the incidence, diagnosis and management of ICPI-associated AKI, and address the controversy surrounding kidney biopsy in diagnosis of ICPI-associated AKI.

## Definition and incidence of ICPI-AKI

The overall incidence of AKI in patients receiving ICPIs is close to 17% (range 7 to 24%), although this estimate reflects both ICPI-related and non-ICPI-related etiologies (14–18). To date, there is no standardized definition of ICPI-AKI, largely due to the varying definitions of ICPI-AKI employed in early studies. Two commonly used grading scales for ICPI-AKI are the National Cancer Institute's Common Terminology Criteria for Adverse Events (CTCAE) and the Kidney Disease: Improving Global Outcomes (KDIGO) consensus criteria. CTCAE defines AKI through comparison to a known baseline or the upper limit of normal whereas KDIGO stratifies AKI according to relative changes in serum creatinine (19, 20). While we prefer KDIGO criteria due to recognition that large shifts in serum creatinine may not break the upper limit of normal especially in cachectic patients, major oncologic societies have based proposed irAE treatment on CTCAE definitions, which may make this a more applicable AKI definition.

Lack of a standardized definition has made it challenging to discern the true incidence of ICPI-AKI. However, one early study, which pooled data from phase II and III clinical trial data published between 2014 and 2015 that included at least 100 patients receiving ICPIs, estimated incidence of ICPI-AKI as approximately 2% (21). Severe AKI (defined as serum creatinine >3 times above baseline, an increase in serum creatinine to a level >4.0 mg/dL, or need for renal replacement therapy) occurred in 0.6% of patients. The incidence of ICPI-AKI rose to ~5% in patients receiving combination ipilimumab plus nivolumab therapy. A more recent meta-analysis including over 11,000 patients receiving PD-1 inhibitors specifically found the pooled incidence of AKI (defined as serum creatinine at least increase >0.3 mg/dL or creatinine at least >1.5–2 times above baseline) to be 2% (22). In another narrative review, the reported incidence with a similar definition of ICPI-AKI was as high as 9.9 to 29% (23).

The median time of onset to ICPI-AKI varies, ranging anywhere from 1 to 12 months depending on the specific ICPI prescribed. One review found that the average onset of AKI was shorter in patients treated with the anti-CTLA-4 agent ipilimumab (6 to 12 weeks), as compared to anti-PD-1 agents pembrolizumab (1 to 12 months) and nivolumab (6 to 12 months) (23). Cortazar et al. reported an average onset of 14 weeks after initiation of therapy (range 6 to 37 weeks) when analyzing both anti-CTLA-4 and anti-PD-1 agents (24). Another multicenter study evaluating over 400 patients diagnosed with ICPI-AKI between 2012 and 2020 found that ICPI-AKI developed at a median of 16 weeks, although notably ~11% of cases occurred over a year after ICPI initiation (25). The prolonged time from ICPI exposure to AKI development may result from the relatively long half-life of these drugs and the prolonged duration of immune system activation. This can make recognizing the cause of AKI difficult.

## Diagnosis of ICPI-AKI

Patients with cancer are predisposed to AKI for a variety of reasons. Current expert opinion suggests evaluation for ICPI-AKI should begin once other more common causes of AKI have been ruled out. These include causes such as volume depletion, acute tubular injury secondary to hemodynamic fluctuations or sepsis, cancer-related urinary tract obstruction, exposure to other known nephrotoxins, or causes related to medical co-morbidities including cardiorenal and hepatorenal syndromes. In kidney biopsy proven cases of ICPI-AKI, acute tubulointerstitial nephritis (ATIN) was the most common manifestation of kidney injury followed by various glomerulopathies, and acute tubular injury (ATI) (21, 26–38).

Potential mechanisms for development of ATIN following ICPI therapy have been described. One theory proposes that the widespread disinhibition of T-cells leads to loss of tolerance to potential haptens, low molecular weight drug compounds that bind to self-proteins, triggering an immune reaction (26). Another hypothesis argues that dual anti-CTLA-4/anti-PD-1 effects synergistically break self-tolerance, releasing tissue-specific self-reactive T cells which express high levels of PD-1 receptor to target self-antigen, predisposing the patient to autoimmune side effects (39). Presence of CD4+ lymphocyte-rich interstitial infiltrates seen on kidney biopsy in cases of ICPI-AKI supports an autoimmune process (40). Other theories exist but are less likely.

Due to this described loss of tolerance, concurrent use of medications that can trigger an immunoallergic response such as proton pump inhibitors (PPIs), non-steroidal anti-inflammatory medications (NSAIDs) and various antibiotics serve as important risk factors in the development of ICPI-AKI. For example, one multicenter study including 138 patients diagnosed with ICPI-AKI found that 54% of patients were also receiving a PPI. Furthermore, 22% of the study patients were taking NSAIDs and 9% were taking an antibiotic associated with ATIN (24). Nearly 50% of patients in a similar large, multicenter cohort were concurrently receiving PPIs at the time of ICPI-AKI diagnosis (25). Frequency of ICPI-AKI increased to approximately 60% when patients reported taking a combination of PPIs, NSAIDs or certain antibiotics. Results from smaller, single-center studies have supported the association between ICPI-AKI and these medications, with PPIs being the more commonly implicated medication (14, 26, 41). A recent study has attempted to stratify incidence of ICPI-AKI with various PPIs, finding that the concurrent use of omeprazole had higher incidence of ICPI-AKI in patients receiving ipilimumab or nivolumab (42). Fortunately, patients who developed ICPI-AKI with concurrent use of ATIN-associated medications appeared to have greater probability of AKI recovery, likely attributable to the cessation of culprit ATIN-associated medications at time of diagnosis (24).

Additional clinical features that appear to predispose patients to developing ICPI-AKI include use of combination immune checkpoint therapy and lower baseline eGFR (defined as eGFR <60 mL/min per 1.73 m^2^) (21, 24, 25). A double-blind randomized clinical trial comparing ipilimumab plus nivolumab compared to ipilimumab alone for untreated melanoma found the incidence of renal related adverse events in three patients treated with combination therapy (*n* = 94; incidence 3%) vs. zero patients treated with ipilimumab alone (*n* = 46) (43). One large multicenter study investigated independent risk factors for ICPI-AKI demonstrating the greatest risk in patients receiving combination anti-CTLA-4 and anti-PD-1/PD-L1 agents (adjusted odds ratio, 3.88; 95% confidence interval, 2.21 to 6.81) (24). In another study comparing 846 patients with (*n* = 429) and without (*n* = 427) ICPI-AKI, older patients and patients with genitourinary cancers were also more likely to develop ICPI-AKI (25).

While these kidney toxicities may be relatively uncommon, existence of other extrarenal irAEs may clue providers to the presence of an underlying ICPI-AKI. One study demonstrated concomitant extrarenal irAEs in 26 of 30 patients (87%), with thyroiditis and colitis being the most commonly associated immune adverse events (14). Another study found a more modest degree of co-occurrence, with extrarenal irAEs appearing in 43% of patients diagnosed with ICPI-AKI (24). In this study, rash, hepatitis and colitis occurring before or during ICPI-AKI were the most common manifestations. In a large retrospective cohort, Gupta et al. reported an adjusted odds ratio of 2.07 in patients with extra-renal irAEs (confidence interval 1.53–2.78) (25).

Certain laboratory findings may suggest ICPI-AKI, although no single feature or combination of features is specific enough to confirm a diagnosis. Studies featuring biopsy-proven ICPI-AKI highlighted the presence of sterile pyuria or white blood cell casts in 33 to 83% of cases (21, 25–27, 41). Presence of hematuria (9–39%), eosinophilia (7–16%) and worsening hypertension (15%) has also been reported to a less significant degree (21, 25, 26, 44). Proteinuria, if present, is generally in the sub-nephrotic range (21, 26, 27). Recent developments in the use of novel urinary cytokine biomarkers IL-9 and TNF-alpha to distinguish ATIN from ATI show promise; however, further work is needed to establish these markers as reliable non-invasive diagnostic tools (45). Additionally, a small study recently revealed that serum CRP and urine retinol binding protein/creatinine ratio may help differentiate ICPI-associated AKI from AKI due to other etiologies (44).

In patients who undergo kidney biopsy, ATIN is the most common histopathologic finding, which typically demonstrates mild to severe interstitial inflammation without glomerular involvement (21, 31, 46). Interstitial infiltrates are typically lymphocyte-predominant with occasional eosinophils and plasma cells (40). Granulomatous features may also be seen (21). Severity of interstitial inflammation on kidney biopsy has not been shown to correlate with severity of AKI. In one case series of metastatic melanoma patients, 25% of kidney biopsy lesions (*n* = 3/12) also demonstrated moderate to severe tubular atrophy and interstitial fibrosis (46).

Glomerular injury has been described in ICPI-AKI, albeit infrequently. In patients with glomerulopathies, minimal change disease, pauci-immune glomerulonephritis, and complement 3 glomerulonephritis are most common (32, 36, 38). One systematic review found that only 17% of pauci-immune glomerulonephritis cases had positive ANCA serologies, indicating most were ANCA-negative vasculitis (32). Case reports of acute immune-complex mediated glomerulonephritis, lupus nephritis, anti-glomerular basement membrane disease and Goodpasture's disease also exist (32–35, 41, 46, 47). Recent studies show that incidence of glomerular injury appears to be higher in patients treated with anti-PD1/anti-PDL1 agents (32, 41).

## Role of kidney biopsy in ICPI-AKI

The role of kidney biopsy in diagnosis of ICPI-AKI is somewhat controversial and currently debated (Table 1). Many providers believe that clinical suspicion coupled with the presence of supporting laboratory data and/or lack of other readily identified cause is enough to make a diagnosis of ICPI-AKI and begin empiric corticosteroid treatment. Similarly, current clinical practice guidelines for oncologists recommend against routine kidney biopsy for diagnostic purposes. The American Society of Clinical Oncology (ASCO) practice guidelines recommend proceeding directly to empiric corticosteroid treatment for Grade ≥2 toxicities (defined as serum creatinine at least 2–3 times above baseline) if other causes of AKI are ruled out (48). The guidelines further state that “reflex kidney biopsy should be discouraged until corticosteroid treatment has been attempted”. The National Comprehensive Cancer Network (NCCN) practice guidelines suggest consideration of kidney biopsy only for Grade ≥3 toxicities (defined as serum creatinine at least >3 times baseline or >4.0 mg/dL) (49). Kidney biopsy is then pursued if a patient fails to respond to corticosteroid treatment to assess for alternative etiologies of AKI.

**Table 1:** Pro and con arguments for kidney biopsy in ICPI-AKI.

**Kidney biopsy**	**Empiric steroids**
•More specific to detect culprit lesion than non-invasive testing •Accurate diagnosis guides appropriate management (including cases of ATIN, glomerular disease, or tubular injury) •Potential to spare patients long and possibly harmful corticosteroid courses •Can continue ICPI if lesion is not irAE •Useful for future research to better understand and treat ICPI-AKI	•Majority of lesions are ATIN which can be treated with corticosteroids •Early initiation of corticosteroids can improve renal recovery •Avoids potential complications of kidney biopsy •Presence of extrarenal irAEs prompts corticosteroid therapy irrespective of kidney lesion

In contrast, a growing number of clinicians support the pursuit of diagnostic kidney biopsy in cases of suspected ICPI-AKI for several key reasons. First, recognized laboratory data (sterile pyuria, urinary leukocyte casts, eosinophilia, etc.) are not consistently present in many cases of biopsy proven ATIN including ICPI-AKI, as evidenced by the wide range of incidences reported in prior studies (21, 26, 27, 41, 50, 51). Second, the histologic information obtained from the biopsy may be instrumental in guiding patient management. For example, identification of non-ICPI-induced kidney lesions such as acute tubular injury on kidney biopsy would prevent unnecessary and potentially harmful corticosteroid exposure and permit continued ICPI therapy. This is an important point as corticosteroids are not a benign treatment. Major side effects include poor glycemic control, weight gain, fluid retention, disruption in mood, and risk of opportunistic infection with prolonged use. One study noted that even an average of 6 days of oral corticosteroids (maximal dose, 40 mg/day) was associated with an increased incidence of diabetes mellitus, sepsis, venous thromboembolism, and fractures (52). Furthermore, a meta-analysis suggested that corticosteroids may also blunt the efficacy of ICPIs in patients with non-small cell lung cancer (53). Premature discontinuation of ICPI therapy without confirmatory testing can negatively impact treatment outcomes by halting a potentially life-saving treatment. A recent retrospective study from Japan comparing non-small cell lung cancer patients who received systemic corticosteroids for anti-PD-1 related irAEs to those who did not develop irAEs or receive corticosteroids for irAEs found that progression free survival was significantly shorter in patients who received systemic corticosteroids (11.7 vs. 16.0 months; *p* = 0.037) (54). However, other studies show no difference in survival (55, 56). Cases of suspected ICPI-related glomerulopathy warrant kidney biopsy to further characterize the lesion and inform prognosis as pathologies such as pauci-immune glomerulonephritis carry greater risk of progression to dialysis dependence or death (32). In light of these arguments, the Society for Immunotherapy of Cancer (SITC) recently released guidelines with the following proposal: “Given the lack of specific clinical features for ICI-related AKI, kidney biopsy should be strongly considered when feasible, particularly when a plausible alternative etiology for AKI exists or urine studies are suggestive of glomerular disease.” (57) We agree with these guidelines and recommend pursuit of diagnostic kidney biopsy in most cases.

In ICPI-AKI patients who require immunosuppressive therapy for extrarenal irAEs, it is reasonable to forego kidney biopsy and continue close monitoring of kidney function. Likewise, in the absence of another identifiable etiology of AKI after thorough evaluation, clinicians should consider proceeding with empiric corticosteroid therapy if there is a contraindication to kidney biopsy such as renal or perirenal infection, severe hypertension, or coagulopathy. Initiation of corticosteroids should not be delayed when there is a strong suspicion for ICPI-induced ATIN (extrarenal irAEs, urinary leukocyte casts), as early initiation of corticosteroids (within 3 days of ICI-AKI occurrence) has been shown to enhance renal recovery (25). If empiric corticosteroids are initiated for ICPI-AKI prior to histopathologic confirmation, a kidney biopsy should be performed in cases where a patient fails to respond to initial treatment or when relapse occurs to assess for alternative etiologies of AKI.

## Management of ICPI-AKI

**Figure 1:**
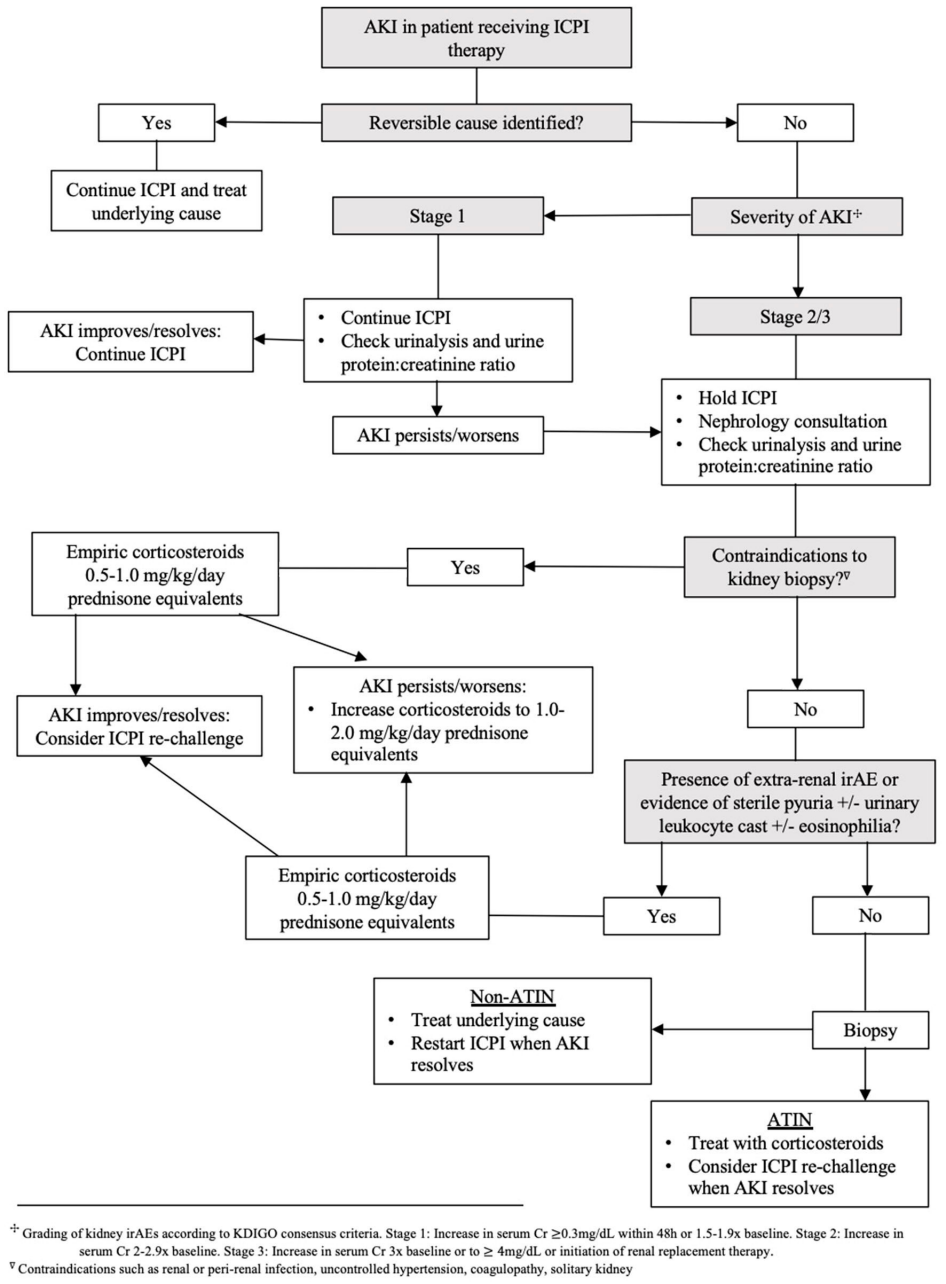
Evaluation and initial management algorithm for suspected ICPI-AKI.

Corticosteroids are the backbone of ICPI-AKI treatment. In one large multicenter study, 103 of 119 patients (87%) with ICPI-AKI who were treated with corticosteroids had partial or complete kidney recovery (24). Management is further dependent on the severity of kidney toxicity as defined by the CTCAE version 5.0 (20). The ASCO and NCCN clinical practice guidelines recommend temporary suspension of ICPI therapy and initiation of 0.5–1 mg/kg/day prednisone equivalents for Grade ≥2 toxicities (48, 49). If there is no improvement or worsening in kidney function, the guidelines propose increasing to 1–2 mg/kg/day prednisone equivalents and permanent discontinuation of ICPI therapy. Addition of further immunosuppression (e.g., mycophenolate mofetil, cyclophosphamide, infliximab or rituximab) can be considered in severe ICPI-AKI. While recommendations for these second-line immunosuppressive regimens are largely based off expert opinion, use of the anti-TNF-alpha agent infliximab has gained traction due to success in treating steroid resistant extrarenal irAEs (58–60). Discovery of high levels of TNF-alpha circulating in patients treated with ICPIs may explain this favorable response to infliximab therapy (30). If and when kidney function improves to Grade 1 toxicity (defined as serum creatinine level increase of >0.3 mg/dL or creatinine 1.5–2 times baseline), it is reasonable to taper corticosteroids over a 4–6 week period with weekly monitoring of renal function. Based on current guidelines and our opinion, Nephrology consultation and strong consideration for kidney biopsy is recommended for all Grade ≥2 toxicities. Management of ICPI-AKI according to different societal guidelines is highlighted in Table 2. Our proposed algorithm for initial evaluation and management of ICPI-AKI is featured in Figure 1. We employed the KDIGO criteria as the most appropriate method to stage AKI to allow both the oncology grading system and KDIGO staging system to be available for the readers.

**Table 2:** Comparison of clinical practice guidelines for management of ICPI nephrotoxicity.

	**ASCO**	**NCCN**	**SITC**
Limited diagnostic work-up	Routine U/A not necessary	Obtain spot urine protein:creatinine ratio	Obtain U/A and spot urine protein:creatinine ratio
Recommendation for kidney biopsy	Proceed directly to corticosteroids if no alternative cause identified	Consider biopsy in G3 toxicities; otherwise, proceed directly to corticosteroids	Consider biopsy if plausible alternative etiologies exist or urine studies suggest glomerular disease
Management	Hold ICPI and start 0.5–1 mg/kg/day prednisone equivalents. Increase to 1–2 mg/kg/day if no improvement. Permanently discontinue in ≥G3 toxicities	Hold ICPI and start 0.5–1 mg/kg/day prednisone equivalents. Increase to 1–2 mg/kg/day if no improvement. Permanently discontinue in ≥G3 toxicities. Add additional immunosuppression if ≥G2 after 1 week	Hold ICPI and biopsy if clinically feasible. Otherwise proceed with corticosteroids (no regimen outlined). Consider addition of infliximab or mycophenolate mofetil after 1 week
Nephrology consultation	Recommended for ≥G2 toxicity	Recommended for ≥G2 toxicity	Recommend for all progressive or persistent G1 and above
ICPI re-challenge	Consider if no recurrence of AKI or chronic renal insufficiency	Consider on resolution of ICPI-AKI to ≤ G1 with or without corticosteroid. For ≥G2, consider rechallenge at least 2 months after holding ICPI	Consider rechallenge in ≥G2 if AKI resolves or is controlled with less than 10mg/day prednisone equivalents

*U/A, urinalysis; ICPI, immune checkpoint inhibitors; AKI, acute kidney injury.*

*Grading of kidney irAEs according to CTCAE version 5.0. G1: Serum Cr >0.3mg/dL increase or 1.5-2x baseline. G2: Serum Cr 2-3x baseline. G3-4: Serum Cr >3x baseline, Cr >6.0mg/dL or need for dialysis.*

*Clinical practice guideline abbreviations: American Society of Clinical Oncology (ASCO); National Comprehensive Cancer Network (NCCN); Society for Immunotherapy of Cancer (SITC)*.

Permanent discontinuation of ICPI therapy can have serious implications on patient outcomes, including progression free survival and/or overall survival. This is especially true in patients who have exhausted other non-ICPI treatments. As such, the decision to rechallenge patients with ICPIs should be explored with thorough review of the risks and benefits. Certain factors may influence the decision to re-challenge patients including clinical circumstances at the time of diagnosis (e.g., timing of irAE onset, presence of other ATIN-associated medications that can be discontinued, or use of combination therapy which may be narrowed to monotherapy). Current ASCO guidelines recommend the consideration of ICPI re-challenge in patients who have improvement in renal function after initial kidney injury (i.e., improvement to Grade 1 toxicity). To support this recommendation, a large cohort study reported ICPI re-challenge in 121 patients with only 20 patients (16.5%) experiencing recurrent ICPI-AKI (25). Median length of time until recurrent ICPI-AKI was about 10 weeks. Importantly, 12 of 20 recovered kidney function with ICPI discontinuation and corticosteroids. In another multicenter study, re-challenge was attempted in 31 patients with 7 patients (23%) developing recurrent ICPI-AKI (24). Of these 7 patients, only one did not recover and suffered permanent kidney injury. Other retrospective studies support resumption of ICPI while patients remain on low-dose immunosuppression (prednisone 5–20 mg/day). In this setting, recurrence of ICPI-AKI was reported in 5–25% of cases (24, 44, 61, 62). It is important to note that while many clinicians choose to maintain low-dose immunosuppression during ICPI re-challenge, current guidelines do not recommend for or against concurrent administration of these medications to improve outcomes. Dose adjustments are not necessary during ICPI re-challenge (48).

## Summary and future directions

ICPI-AKI is an increasingly recognized cause of kidney injury in patients with cancer. ATIN is the most common histopathologic finding on kidney biopsy although other kidney lesions may be seen. Medications known to cause ATIN such as PPIs, NSAIDs and certain antibiotics can increase the risk of ICPI-AKI, possibly through host immune system loss of tolerance to these potential haptens. Treatment with combination immunotherapy also carries greater risk for nephrotoxicity. Sterile pyuria, white blood cell casts, and eosinophilia have been reported in ICPI-AKI, although these clinical features are not reliable enough for diagnosis. While the role of kidney biopsy in diagnosis of ICPI-AKI is currently debated, a growing number of clinicians support the use of kidney biopsy to confirm cases of suspected ICPI-AKI and inform treatment choices. Timely pursuit of kidney biopsy may help clinicians avoid potentially harmful corticosteroids and allow continuation of immunotherapies in situations of non-ICPI-related AKI.

As indications for ICPI use expand, future work should focus on identification of both sensitive and specific non-invasive diagnostic markers for early detection of ICPI-AKI (primarily ATIN). A recent externally validated diagnostic model for the prediction of ATIN using serum and urine markers shows modest area under the receiver operating characteristics curve (AUC 0.73; confidence interval 0.64–0.81). A model controlling for blood eosinophils and dipstick leukocytes and protein demonstrated an AUC of 0.84 (confidence interval 0.76–0.91) for both biomarkers, suggesting potential utility for non-invasive diagnosis (63). Further investigations would be necessary to validate use of these biomarkers in the ICPI population. Given the potential life-threatening consequences of permanent ICPI discontinuation, more data is needed to discern which patients may be safely re-challenged and if continued low-dose immunosuppression during ICPI re-challenge decreases the risk of recurrent ICPI-AKI. Finally, greater understanding of the mechanisms driving ICPI-induced glomerular diseases could inform appropriate management of these rarer kidney toxicities.

## Author contributions

EM made contributions to conception of the work and drafted the manuscript. MP made contributions to conception of the work and to the drafting and revision of the manuscript. Both authors contributed to the article and approved the submitted version.

## Conflict of interest

The authors declare that the research was conducted in the absence of any commercial or financial relationships that could be construed as a potential conflict of interest.

## Publisher's note

All claims expressed in this article are solely those of the authors and do not necessarily represent those of their affiliated organizations, or those of the publisher, the editors and the reviewers. Any product that may be evaluated in this article, or claim that may be made by its manufacturer, is not guaranteed or endorsed by the publisher.
